# Convergent Evolution: Self-Assembly of Small Molecule, Polymeric, and Inorganic Contrast Agents toward Advanced MRI

**DOI:** 10.1021/jacs.4c11767

**Published:** 2025-12-17

**Authors:** Fuqiang Chang, Gemma-Louise Davies

**Affiliations:** School of Chemistry, 1724 University of Birmingham, Edgbaston, Birmingham B15 2TT, U.K.

## Abstract

Magnetic resonance imaging (MRI) is a non-invasive technique providing detailed anatomical, physiological, and molecular information. Contrast agents (CAs), such as gadolinium-based chelates, enhance differentiation between normal and abnormal tissues, highlight blood vessels, and detect tumors, inflammation, or other conditions, to aid clinical diagnostics. Although small-molecule chelate-based CAs have been used clinically for several years, extensive research efforts have been directed toward enhancing CA properties, ranging from boosting their contrast-to-noise ratio to developing responsive agents, through careful design. This perspective examines the convergent (not chronological) evolution of different classes of CAs: small-molecule complexes, polymeric chelates, and inorganic nanoparticles. We outline how research across these types of CAs has led to self-assembled species showcasing superior MRI performance compared to traditional agents. Despite different mechanisms driving the two main MRI CA classes (positive, T_1_ and negative, T_2_), similar strategies have been exploited to drive changes in structure, active-species interaction, and modulation of water access. Self-assembled CAs not only demonstrate considerably slower tumbling rates and enhanced water interactions, which mechanistically boost signal, but also generally manifest improved biodistribution, effective passive targeting to tumor sites, and tuneable clearance pathways when utilized as delivery carriers. These agents further exhibit promise for switchable and active in vivo behavior, valuable in clinical diagnostics. However, despite significant advances, translation to clinic remains a bottleneck due to the complex relationship between mechanistic parameters which define MRI and how they ultimately behave in the body. As such, much work remains, and this perspective aims to inspire by demonstrating how the “old generation” of CAs could evolve to address these challenges.

## Graphical abstract

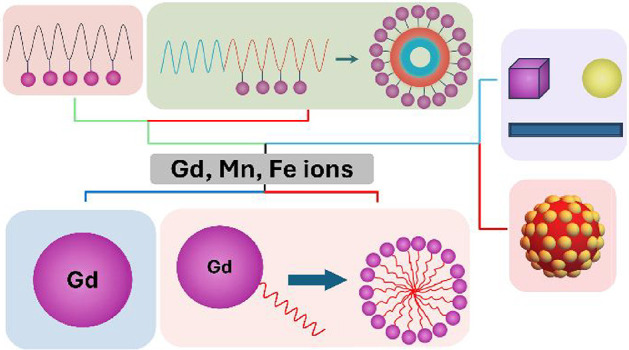

## 1 Introduction

Magnetic Resonance Imaging (MRI) is a non-invasive imaging technique offering detailed insights into anatomical, physiological, and molecular information in living organisms, with the advantages of deep penetration and variable spatial resolution without ionizing radiation.^1^ In MRI, when subjected to an external magnetic field, magnetic nuclei such as ^1^H, ^13^C, ^31^P, ^17^O, ^19^F, ^23^Na, and ^129^Xe undergo nuclear magnetic resonance through radiofrequency pulse stimulation.^2^ Among these nuclei, ^1^H is extensively studied due to its high gyromagnetic ratio and abundant presence in biological tissues. Comprehensively reviewed in numerous articles,^3–7^ MRI signal is modulated by the relaxation dynamics of these ^1^H spins, which vary depending on their molecular environments – ranging from tumors to tissues, fat and blood – exhibiting distinct relaxation times. MRI predominantly utilizes two relaxation processes, longitudinal and transverse relaxation, simplified as T_1_ and T_2_ relaxation, respectively, to produce detailed anatomical images of use in clinical diagnostics.^4,8^

In the late 1940s, Bloembergen, Purcell and Pound described how paramagnetic ions significantly enhance nuclear spin relaxation, with their theory explaining how relaxation times are influenced by molecular motion and interactions with paramagnetic centers.^9^ It was not until the 1970s that this effect was leveraged for MRI; Lauterbur pioneered the use of magnetic field gradients to spatially encode NMR signals, enabling the first MRI images of internal structures in the body. This breakthrough transformed NMR from a spectroscopic technique into an imaging modality, laying the foundation for modern MRI.^10–12^ Nowadays, gadolinium paramagnetic species (used in their chelated form to avoid toxicity resulting from free Gd^3+^ ions) are extensively used as T_1_ contrast agents (CAs) in MRI.^13^ Despite Gd^3+^-based CAs like Dotarem remaining the most popularly used agent clinically in modern medicine, small-molecule chelates possess drawbacks such as low contrast-to-noise ratios and reduced efficiency compared to free ions. This necessitates higher doses, increasing the risk of adverse side effects like nephrogenic systemic fibrosis and gadolinium deposition in multiple organs, including the brain, kidneys, liver, and bones, which raises concerns about the long-term health impacts.^14^ Thus, the development of probes with enhanced relaxivity (not only in terms of increased contrast-to-noise capabilities but also potential diagnostic utility) is imperative for advancing MRI and has therefore been the subject of intense investigation for several decades.

Herein, we examine self-assembly design strategies of small-molecule, polymeric and inorganic particle-based CAs, showcasing the convergent evolution of similar mechanistic design features, and highlighting pioneers and exciting new avenues in this consistently popular research area. We further discuss the limitations of clinical application of promising new materials. Notably, our goal in this manuscript is not to comprehensively describe the optimization of all parameters for a single type of MRI CA, but rather to provide a broader overview of the potential of self-assembly to enhance the performance of MRI CAs. While the importance of optimizing various factors for a singular CA is key in the design of new high relaxivity CAs (considering water exchange, tumbling rate, electron spin relaxation, and other parameters for small-molecule CAs; and the influence of size, shape, crystallinity, surface modification, or element doping for magnetic inorganic nanoparticles), we have intentionally limited discussion of these aspects, as they have been extensively covered in prior reviews.^1,4,6,15–18^ As a non-comprehensive review of the field, we have selected key example publications which serve as representative cases for the synthesis strategies, development progress, and advancements relevant to the growing interest in self-assembled constructs for MRI.

## 2 Early Evolution of MRI CAs: From Small Molecules, to Polymeric, and Inorganic Nanoparticles and Clusters

The development of Solomon-Bloembergen-Morgan (SBM) theory allowed a clear understanding of relaxivity (*r*_1_, a quantitation of MRI signal enhancement efficacy) of T_1_ CAs, permitting prediction of behavior. Relaxivity is primarily determined by four key parameters: the number of inner-sphere water molecules (*q*), the mean residence lifetime of the bound water molecule (τ_m_), the electron spin relaxation, and the tumbling rate of the complex in solution (1/τ_R_), where τ_R_ represents the rotational correlation time.^3^ A higher *q* value typically results in a higher *r*_1_. However, increasing *q* usually reduces the coordination number of the stabilizing ligand or uses more flexible chelating arms, compromising the kinetic and thermodynamic stability of the complex.^13,19^ Consequently, much effort in enhancing MRI CA efficiency has focused on optimizing τ_m_ and τ_R_. Particularly at low to moderate magnetic field strengths (≤3 T), increasing τ_R_ has a more pronounced effect on improving relaxivity compared to reducing τ_m_.^20^ Therefore, adjusting τ_R_ is a commonly explored and crucial factor for achieving high relaxivity in most CAs.^6,21,22^

Increasing the molecular weight of the complex to slow down the tumbling rate is a straightforward method to enhance relaxivity and has been widely applied; appending paramagnetic chelates to polymers or macromolecules are an obvious choice to achieve this (Scheme 1). Larger complexes often demonstrate slower clearance and enhanced accumulation and retention in vivo compared to low-molecular weight small molecules, further enhancing the observed MR signal.^23,24^ Higher molecular weight systems further facilitate the integration of multiple complexes and targeting groups into one system, boosting the concentration of CAs in targeted regions. The properties of the polymer itself can significantly impact the relaxivity enhancement for polymeric CAs. For instance, polymers with rigid backbones or linkers can more effectively impede rotational motion of the complex, leading to a more significant increase in *r*_1_ compared to more flexible polymers; dendrimers or cross-linked polymers inherently possess greater rigidity than linear analogues, limiting freedom for conjugated chelates to rotate.^25–29^ Furthermore, cross-linked and dendritic structures rotate isotropically, whereas linear oligomers rotate more rapidly about their long axis, which can restrict their relaxivity.

**Scheme 1:**
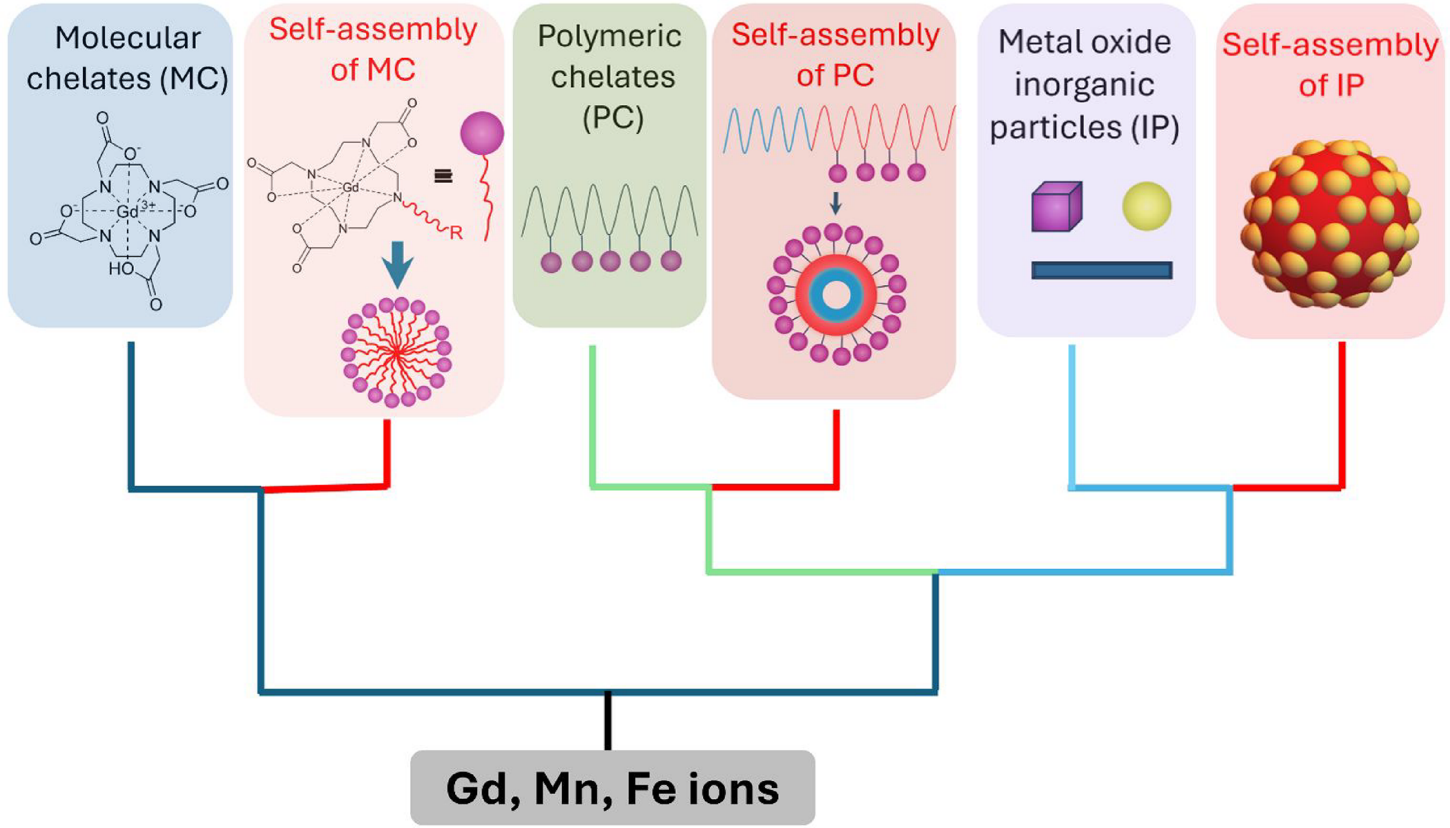
A phylogenetic tree of MRI CAs shows how CAs have developed from small-molecule complexes to polymeric chelates to inorganic nanoparticles, as well as the evolution of self-Assembly of the three types of CAs (red lines and boxes)

Exploiting similar mechanistic advantages, Gd^3+^-chelates can be appended to inorganic materials to boost contrast. This approach has been extensively demonstrated for materials such as carbon nanotubes,^30^ graphene,^31,32^ nanodiamonds,^33,34^ and metal-based systems like gold.^35,36^ For example, nanodiamonds (ND) covalently immobilized with Gd^3+^-chelates form small Gd^3+^-ND clusters, whose increased bulk contribute to significant relaxivity enhancements compared to Gd^3+^-chelates alone.^37^ An often-used host particle is silica (usually in the form of mesoporous silica nanoparticles), where grafting Dotarem-type species has been shown to enhance relaxivity due to changes in τ_m_ and τ_R_, influenced by the particle bulk, rotation around the covalent linkage, and the unique dynamics of the porous channels.^38–40^ Notably, when immobilized onto larger scaffolds (such as polymers, dendrimers, and inorganic particles), 1,4,7,10-tetraazacyclododecane-1,4,7-triacetic acid (DO3A)-monoamide-type complexes are often used, due to their ease of surface grafting; however the lowered kinetic stability of these (compared to 1,4,7,10-tetraazacyclododecane-1,4,7,10-tetraacetic acid (DOTA), for example), could lead to dissociation and release of free Gd^3+^.^4,41^ This is undesirable due to potential health risks associated with long-term accumulation, and therefore caution in selection of grafting compound must be employed, regardless of the final conjugate design. Although traditional Gd^3+^-chelates have been explored for many years as T_1_ CAs, Mn and Fe based small-molecule CAs are becoming more popular due to their exceptional biocompatibility. Unlike Gd, which can pose challenges related to toxicity and retention in the body, Mn and Fe are essential nutrients in the body with natural homeostatic pathways, which can in principle mitigate long-term retention issues observed with Gd^3+^. Thus, their small-molecule complexes can provide effective contrast enhancement while minimizing potential adverse effects.^42–44^

New types of inorganic nanomaterials, like Gd_2_O_3_ and MnO_2_, have also been tailored for use as MRI CAs, showcasing exceptional T_1_ relaxivity augmentation due to excellent water accessibility to the high paramagnetic surface areas.^45,46^ The small size of Gd_2_O_3_ nanomaterials allows them to pass through tumor vascular pores, and their renal clearance helps reduce exposure to heavy metal Gd^3+^ ions.

On the other hand, superparamagnetic iron oxide nanoparticles (SPIONs), typically 5–20 nm in core diameter (up to ∼100 nm in hydrodynamic size), are widely used as T_2_ contrast agents. Their relaxation enhancement primarily arises from local magnetic field inhomogeneities that induce dephasing of nearby ^1^H spins, with diffusional correlation times (τ_D_) influencing the extent of this effect. However, SPIONs can exhibit limited T_2_ relaxivity, resulting in reduced magnetic contrast effectiveness and limited imaging capabilities in deeper tissues. To overcome this challenge and enhance T_2_-weighted MRI signals, various strategies have been explored.^47^ These include increasing the size of iron oxide nanoparticles, enhancing crystallinity, and introducing doping elements to boost saturation magnetization values, or altering particle shapes from isotropic spheres to anisotropic ones with larger aspect ratios, to augment the effective radius of the magnetic particles.^17,48^ Yet, the improvement in T_2_ signal enhancement achieved through these methods is limited, and enlarging nanoparticle size can trigger a transition from superparamagnetic to ferromagnetic behavior, causing colloidal instability due to dipole–dipole interactions.^49,50^ Alternatively, multicore aggregates, or clusters, of SPIONs have been developed, exhibiting improved saturation magnetization values, strong dipole–dipole interactions, and enhanced colloidal stability compared to dispersed individual nanoparticles, while maintaining their superparamagnetic properties. Consequently, these multicore aggregates dramatically enhance T_2_ relaxivity compared to single SPIONs.^1,51,52^ More recently, ultrasmall SPIONs (<5 nm) have been identified as effective T_1_ CAs, benefiting from enhanced signal due to excellent water interaction with the surface iron species.^53–55^

Collectively, these developments highlight how early design strategies exploiting mechanisms such as covalent conjugation, and formulation-induced aggregation, laid the groundwork for more sophisticated, controllable architectures. Building on these foundational principles, more advances are shifting toward the intentional design of self-assembled systems, whose structure and dynamics can impact MRI performance.

## 3 Convergent Evolution: Engineered Self-Assembly

What is clear from early efforts in this domain is that careful consideration of CA design features, whether small-molecule, polymeric or inorganic particles, is vital in achieving contrast enhancements without simply increasing dosages – a desirable clinical outcome. An understanding of mechanisms which define relaxation enhancement (e.g., through SBM equations) is key to achieving boosted signal, and significant efforts in recent years have exploited established mechanistic parameters to boost signal, using different and innovative styles of chemical or physical assembly or interactions (Scheme 1).

Assembly refers broadly to the process by which individual components form larger structures, either spontaneously through non-covalent interactions or under external forces. In this perspective, we specifically focus on self-assembly, which we define as the spontaneous organization of components containing or associated with MRI contrast agents into ordered structures driven primarily by non-covalent interactions. The self-assembly phenomenon is observed across various length scales, and has garnered considerable interest in the fields of chemistry and materials science.^56^ Engineered assembly of molecules or nanoparticles offers a straightforward approach to achieving unique properties that are not inherent in either the individual components or bulk materials.^57^ Indeed, there are numerous fields which already take advantage of self-assembly for improved properties. Self-assembled nanoassemblies have gained increasing prominence as demonstrated by examples such as aggregation-induced emission leading to enhanced fluorescent signals, aggregation of positron emission tomography (PET) tracers enhancing PET signals, and the assembly of up-conversion nanocrystals exhibiting boosted light-converting emission.^58–60^ Considering the mechanisms which define MRI contrast, the self-assembly of MRI CAs holds immense promise for developing multifunctional, bioresponsive nanoplatforms aimed at enhancing MRI diagnostic and imaging capabilities, a concept that has gained traction since the early 2000s.^61–64^ To achieve this, unique and innovative strategies have been developed. Notably, this perspective primarily focuses on CAs where the contrast moiety is integrated into the initial building blocks prior to assembly. Unlike a passive payload attached postassembly, this agent is an integral building block. Its physicochemical properties, such as its charge, hydrophobicity, and coordination geometry, are deliberately engineered to drive and stabilize the self-assembly process. This allows the final nanostructure’s form and function to be encoded at the molecular level. Using “convergent evolution” as an analytical metaphor which is not chronological, this perspective highlights that, despite their chemical and structural diversity, different classes of MRI contrast agents have independently adopted self-assembly as a shared adaptive strategy to enhance MRI relaxivity, stability, and targeting capability. Considering these developments through this lens reveals common functional motifs and unifying design principles that transcend traditional material-based classifications.

One important example is the supramolecular self-assembly of small-molecule CAs, which offers an efficient approach for integrating a large payload of chelates while effectively prolonging the tumbling rate.^65–68^ This assembly relies on various non-covalent bonds, including van der Waals interactions, coordination bonds, hydrophobic/hydrophilic interactions, electrostatic interactions and hydrogen bonds. Importantly, the mechanisms driving MRI signal enhancement through this assembly method are unlikely to result solely from increased rotational behavior due to larger size. The non-covalent interactions extend beyond the structures themselves, and could also influence water access to the MR-active metal centers, water exchange and water diffusion.^69^ For example, Chuburu et al. developed nanohydrogels using the assembly between Gd^3+^-complex and two hydrophilic polymers, chitosan and hyaluronate.^70^ The assembled agents demonstrated a per-Gd^3+^ *r*_1_ relaxivity as high as 100 mM^–1^ s^–1^ under clinical conditions (30 MHz, 37 °C), representing a 23-fold enhancement compared to the free, nonassembled Gd^3+^- chelate. The authors attributed this performance to a “second sphere” effect, where the hydrogel entraps both the Gd^3+^-chelates and a large network of water molecules, creating a highly hydrated environment that drastically improves proton exchange. In another study, Botta et al. reported the assembly of a host–guest adduct between dendrimeric β-cyclodextrin and a Gd^3+^-chelate.^71^ The data shows a clear relaxivity jump upon assembly. The pre-assembly *r*_1_ relaxivity values for the ‘free’ Gd^3+^-dendrimer were 16.7 mM^–1^ s^–1^ at 20 MHz and 25 °C. Upon complex formation, the relaxivity increased by approximately 70% per gadolinium center, with the authors attributing this enhancement to the formation of a rigid, compact structure where high steric hindrance severely limits local rotational flexibility, resulting in a very long effective rotational correlation time (high coupling between global and local rotation). In contrast, T_2_ CAs, including iron oxides, MnO, and Gd_2_O_3_ nanoparticles, rely primarily on the generation of local magnetic field inhomogeneities. Assembly of individual nanoparticles into clusters or networks amplifies these susceptibility effects, thereby significantly enhancing transverse relaxation efficiency.

Here we select a series of examples which exemplify specific approaches to engineering self-assembled molecules, with examples showing improvements in relaxivities compared to nonassembled counterparts.

### 3.1 Self-Assembly of Amphiphilic Small-Molecule Chelators through π-π Stacking and Hydrophobic Interactions

For small-molecule paramagnetic chelates, which typically exhibit a short rotational correlation time, self-assembly can lead to a transformative process, resulting in larger supramolecular particles (distinct from the designed polymer described earlier) with slower rotation rates (Figure 1a).^72,73^ An interesting route to straightforward self-assembly is utilizing π-π stacking and hydrophobic interactions among adjacent hydrophobic segments of small-molecule chelators, with this approach being used to produce both T_1_ and T_2_ CAs.

**Figure 1:**
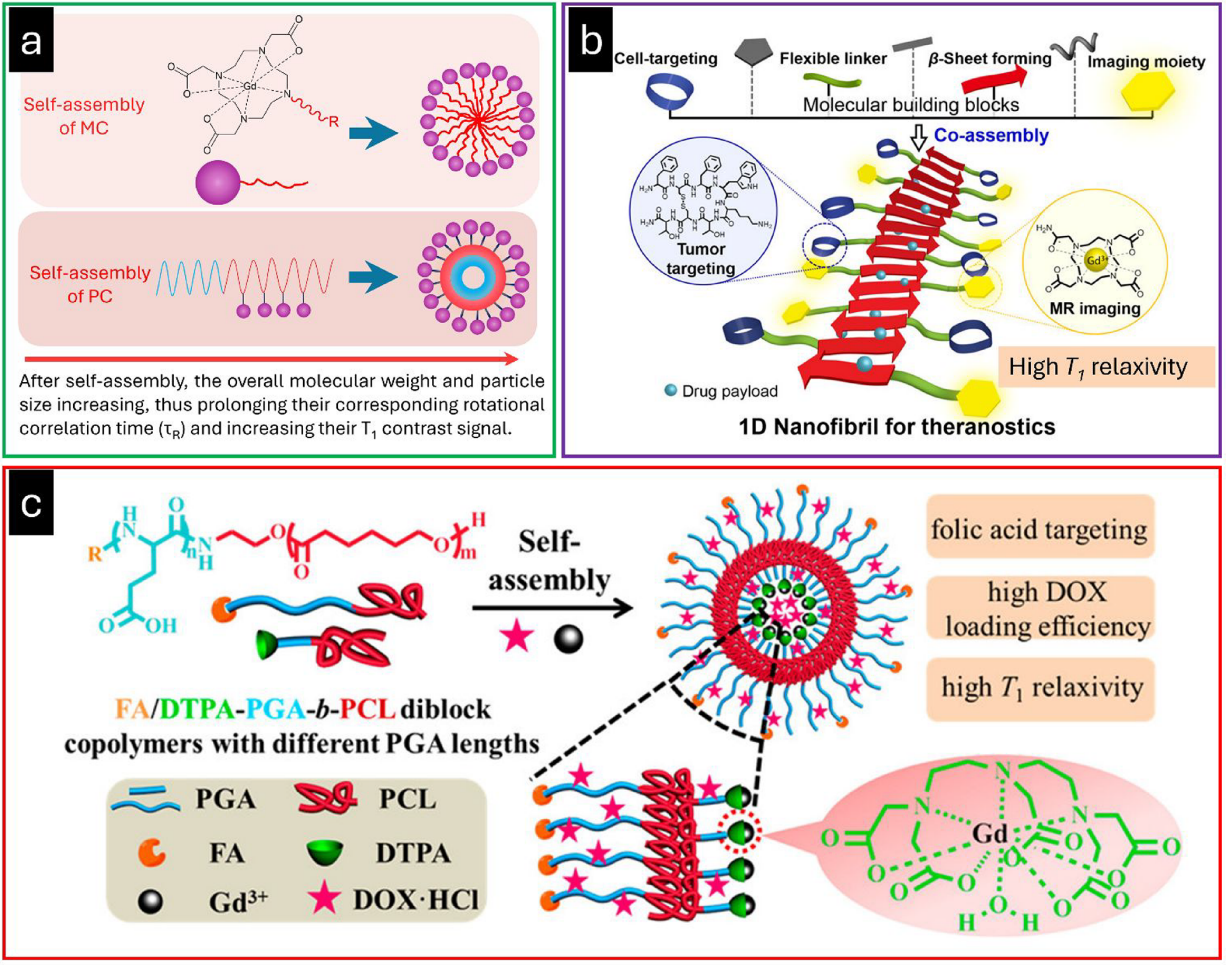
(a) Through self-assembly, small-molecule chelates and polymeric chelates feature large molecular weights and particle sizes, which significantly prolong their corresponding rotational correlation time (τ_R_) and increase their T_1_ contrast signal. (b) Schematic illustration of 1D supramolecular nanoplatform prepared by coassembly of functional peptide amphiphiles. Adapted with permission from ref 73, copyright 2016 American Chemical Society. (c) Illustration of multifunctional asymmetrical polymer vesicles for ultrasensitive T_1_ MRI and effective cancer targeted drug delivery. Blue: biodegradable hydrophilic poly­(l-glutamic acid) (PGA) chains with different chain lengths. Red: biocompatible and biodegradable poly­(ε-caprolactone) (PCL). Orange: tumor-targeting group, folic acid (FA). Green: chelating agent, diethylenetriaminepentacetatic acid (DTPA). Pink: doxorubicin hydrochloride (DOX·HCl). Black: T_1_ CA Gd^3+^. Adapted with permission from ref 74, copy right 2015 American Chemical Society.

Among the small-molecule chelators that leverage this strategy, texaphyrins, which are pentaaza Schiff base macrocycles related to porphyrins, offer a distinctive feature to facilitate this, possessing an “expanded” 5-coordination pocket for metal binding. Zheng et al. have detailed a one-pot synthesis and self-assembly technique for creating Mn^2+^-texaphyrin-phospholipids, enabling easy self-assembly into Mn-nanotexaphyrin nanoparticles. The resulting 100 nm nanoparticles exhibited enhanced *r*_2_ contrast (13.6 mM^–1^ s^–1^ at 7.0 T) compared to individual monomers, which was attributed in the original report to increased effective magnetization and slower tumbling of the nanostructures, although this was not directly proved and care needs to be taken in this interpretation due to the physiological measurement conditions limiting the potential to reach saturation.^72^

In the pursuit of creating multifunctional nanoassemblies, Lee et al. devised a series of peptide amphiphiles (PAs) tailored with either octreotide (a somatostatin analogue for tumor-targeting) or a paramagnetic metal ion Gd^3+^-chelating agent, DOTA, for MRI purposes (Figure 1b).^73^ These two types of PAs were coassembled to seamlessly integrate each PA’s unique functionality, with the heightened hydrophobicity of the peptides inducing a morphological shift from conventional to helical fibrils. The resulting one-dimensional nanoaggregates can successfully facilitate intracellular delivery, thereby enhancing MRI (*r*_1_ reaches as high as 19.5 mM^–1^ s^–1^ at 4.7 T, 25 °C). Improvements are attributed to the formation of fibrils, with slight variations in structure showing small differences in enhancement. This implies that overall size (and reduced global tumbling) is not the only important parameter. Although not directly evidenced in this work, the Gd^3+^ entrapment within the fibrils may also influence the local rotational behavior, indicating that π-π stacking and hydrophobic interactions are promising strategies in supramolecular chemistry. Hydrophilic chelators like DOTA can be made amphiphilic by grafting hydrophobic moieties to the side arms of DOTA, enabling self-assembly. While significant progress has been made in understanding the role of π-π stacking and hydrophobic forces, challenges remain in controlling the size, shape, and stability of assemblies, as well as in overcoming complex synthesis and scalability issues.

### 3.2 Self-Assembly of Metal–Organic Frameworks/Particles

Metal–organic frameworks (MOFs) represent a class of porous hybrid solids formed through the self-organization of metal ions or clusters and organic polydentate bridging linkers.^75^ MOFs exhibit customizable characteristics such as specific surface areas, porosity, and pore diameters, coupled with high crystallinity.^76^ The precise tuning and combination of metal ions and organic ligands enables tailored control over MOF morphology, structure, and functionality, driving extensive exploration across diverse scientific disciplines. There has been a flurry of activity around paramagnetic element-based MOFs which have demonstrated potential as MRI CAs in recent years.^77–79^ For example, Zhao et al. developed a nanoagent loaded with two drugs within Mn–Co MOFs, using a straightforward one-pot self-assembly strategy.^80^ They engineered defects in the MOFs, originating from missing linkers in the MOF network, to achieve a high loading capacity for two drugs, Chlorin e6 and artesunate. The resulting MOF, with a high-spin Mn6-vacancy moiety,^81^ functioned as a T_1_-MRI CA, with enhanced in vivo T_1_-MR signals 24 h postinjection into 4T1 tumor-bearing mice.

Through meticulous design of the structure and composition of assembled nanoparticles, it is possible to create multispecies MOF-based CAs with unique characteristics. An ideal carefully designed example is by Cheon et al., who developed a dual-mode artifact filtering nanoparticle imaging agent (AFIA). It comprised an inorganic core–shell structure consisting of two magnetic components: a superparamagnetic Fe_3_O_4_ nanoparticle core, SiO_2_ as a separating layer, and a paramagnetic Mn-MOF shell ([ImH]­[Mn­(BTC)­(H_2_O)], where ImH = protonated imidazole and BTC = 1,3,5-benzenetricarboxylate). By incorporating a SiO_2_ separating layer, a rigid distance is maintained between the two magnetic materials, controlling the magnetic coupling between them and hence enabling simultaneous high T_1_ and T_2_ contrast effects, improving the accuracy of imaging. The composites exhibited substantial MRI contrast enhancements in both T_1_ and T_2_ images, with relaxivity values at 3.0 T (*r*_1_ = 8.2 mM^–1^ s^–1^, *r*_2_ = 238.4 mM^–1^ s^–1^) more than two times higher than Magnevist (*r*_1_ = 3.8 mM^–1^ s^–1^) and Feridex (*r*_2_ = 109.4 mM^–1^ s^–1^).^82^ The enhanced T_1_ contrast effects of this AFIA are primarily attributed to the structural advantages of the MOF, particularly those associated with the 2-D layered [ImH]­[Mn­(BTC)­(H_2_O)]. A protonated imidazole (ImH^+^), serving as a charge compensator, is situated between the 2D layers, facilitating easy access of water molecules to the Mn^2+^ ions inside the MOF. This configuration promotes effective exchange with inner-sphere water molecules, of vital importance in successful *r*_1_ signal improvement. The magnetic coupling between the two materials (superparamagnetic Fe_3_O_4_ core and paramagnetic Mn-MOF shell) further simultaneously improved both T_1_ and T_2_ contrast performance.

The diverse structures and large number of different materials which can be combined to form MOFs makes them intriguing in the design of MRI CAs, and multimodal probes.^83,84^ Their porosity and tunable pore size offers opportunities to retain and optimize water accessibility and exchange, although control over the crucial parameters which dictate MRI signal enhancement will require significant research investment. Despite their potential, the synthesis and use of MOFs for MRI remains largely unexplored.

### 3.3 Self-Assembly through Macrocyclic Host–Guest Interactions

Host–guest interactions are common in supramolecular chemistry, where larger entities serve as host molecules, often possessing a cavity, with smaller guest species which assemble within this cavity. In the field of MRI nanomaterials, Mn-porphyrin derived molecules have been employed to spontaneously assemble with cyclodextrins through such host–guest interactions into nanoparticles (the *r*_1_ values of porphyrin derived nanoassemblies can reach up to 22.21 mM^–1^ s^–1^ at 3.0 T, room temperature).^85–87^ Taking this one step further, assembly of such host–guest interactions into larger composite particles can facilitate improved imaging properties as well as multifunctionality. β-cyclodextrins are well-established in the literature for behaving as host species. In an interesting study incorporating MRI active units as well as targeting moieties, Lu et al., explored host β-cyclodextrins (βCD) linked to polyhedral oligomeric silsesquioxane (POSS) nanoglobules. Adamantane-appended guest units included an adamantane-modified macrocyclic Gd^3+^ (Ad-(DOTA-Gd)) CA, a cyclic RGDfK peptide targeting ligand with a polyethylenegylcol spacer (Ad-PEG-cRGD), and a fluorescent probe (Ad-Cy5.0) as the guest species. Upon assembly (cRGD-POSS-βCD-(DOTA-Gd)), the targeted host–guest nanoglobular CA demonstrated a specific binding affinity to αvβ3 integrin present in malignant 4T1 breast tumors.^88^ This specialized agent delivered superior contrast enhancement compared to its non-targeted counterpart; the *r*_1_ relaxivity of Ad-(DOTA-Gd) alone measured 3.17 mM^–1^ s^–1^ (at 1.5 T, 37 °C), comparable to reported values for clinical small molecular CAs. Upon complexation with βCD to form βCD-(DOTA-Gd), the *r*_1_ relaxivity increased to 6.36 mM^–1^ s^–1^. Further complexing Ad-(DOTA-Gd) with POSS-βCD to form POSS-βCD-(DOTA-Gd) raised the *r*_1_ value to 9.50 mM^–1^ s^–1^. Although relatively small increases in signal enhancement, this pioneering study demonstrated that the non-covalent binding of small molecular Gd^3+^ chelates to macromolecules or nanoparticles through host–guest interactions increases relaxivity by achieving a prolonged rotational correlation time from the large-sized complexes, coupled with increased rigidity.

The dynamic self-assembly via host–guest interactions offers precise and reversible control, presenting a promising strategy for developing advanced MRI CAs. Stimuli-responsive behaviors inherent to host–guest chemistry can further enhance CA performance by enabling targeted assembly and disassembly under specific physiological conditions. However, this approach remains largely unexplored, with substantial potential yet to be realized. Future research could draw inspiration from supramolecular polymers constructed through orthogonal self-assembly involving host–guest and metal–ligand interactions,^89^ as well as from supramolecular prodrugs based on host–guest chemistry.^90^

## 4 Developing Strategies for the Self-Assembly of Polymeric MRI CAs

Similar to small-molecule CA nanoassemblies, assembled particles of polymeric CAs also exhibit high local CA concentration, extended circulation times, and prolonged rotational correlation times, although the diverse design and composition of polymers could allow wider exploitation of relaxation mechanisms. Compared to the self-assembly of small-molecule CAs, the assembly of polymeric CAs offers a more diverse array of strategies. This versatility is attributed to the various types of polymers available, including linear, branched, cross-linked, and dendrimer polymers. Additionally, polymers can be classified based on their structure, such as homopolymers, block copolymers, alternative copolymers, and random copolymers. Furthermore, the hydrophobicity or charged states of polymers, ranging from hydrophilic and hydrophobic to amphiphilic, positively- and negatively charged polyelectrolytes, provide additional dimensions for tailoring assemblies.^91–93^ In the context of MRI, polymer self-assembly provides potential for signal boosts based on the formation of water pools that increase water accessibility and enhance paramagnetic interactions, alongside traditional rotational correlation improvements resulting from increased tumbling of the bulky assembled structures. For example, Botta et al. reported that the macrocyclic complex [Gd­(DOTP)]^5–^, which lacks directly coordinated water molecules (*q* = 0), exhibited remarkably high relaxivity when assembled within or employed as a cross-linker in nanogels. An exceptional relaxivity of 78.0 mM^–1^ s^–1^ at 20 MHz and 298 K, nearly 20 times greater than that of the free complex, was observed, defying the typical behavior of *q* = 0 Gd^3+^-complexes.^100^ This enhancement was attributed to a strong second-sphere contribution arising from water molecules within the hydrogel matrix that form stable and long-lived hydrogen bond networks with the [Gd­(DOTP)]^5–^ complex. The nanogel environment effectively creates localized water pools with restricted diffusion and prolonged interactions between water and the complex, which facilitate efficient proton relaxation. A similar confinement-driven enhancement was also observed for Gd-DTPA in nanogels, where both the slowed molecular tumbling and the altered water dynamics within these local water pools synergistically amplify relaxivity.^101^ Together, these findings highlight that controlling the local water pool structure within confined assemblies represents a powerful strategy for optimizing MRI contrast performance.

Given the diverse design possibilities of polymeric CAs, various self-assembly strategies have been explored to optimize their performance for MRI applications. In particular, self-assembly driven by electrostatic interactions, amphiphilic properties, and biomolecular assistance (e.g., peptides, proteins, and DNA) emerge as the most versatile and controllable approaches for tailoring particle size, stability, and relaxivity. Therefore, this article focuses on these key strategies to harness the full potential of polymeric CAs in MRI contrast enhancement.

### 4.1 Self-Assembly of Polyelectrolytes through Electrostatic Interaction

Electrostatic interactions, ubiquitous in nature as non-covalent forces, play a crucial role in shaping the structure, behavior, and functionality of biomolecules. They govern processes such as protein folding, conformational shifts induced by pH changes, and molecular recognition.^94^ Moreover, leveraging electrostatic interactions for the self-assembly of basic building blocks finds extensive utility in creating intricate supramolecular structures.^95^ In the realm of MRI CAs, the highly hydrophilic and charged nature of polyelectrolytes makes them attractive to ensure maintained, and enhanced, MRI signal improvement. This has been previously exploited to produce stable colloids of use in MRI,^51,52^ however their polyelectrolyte assembly capabilities hold promise in further elevating their CA potential.

Such assembly has been demonstrated by Yi et al., who showed that the negatively charged 4,4′,4″,4‴-(porphine-5,10,15,20-tetrayl) tetrakis­(benzoic acid) (TCPP) not only assembled with the positively charged polyethylenimine (PEI) to form supramolecular nanoparticles through non-covalent electrostatic interactions, but also served as a chelator for Gd^3+^ ions.^96^ They found that gadolinium porphyrin (Gd-TCPP) efficiently integrated into uniform supramolecular nanoparticles (referred to as Gd-PNPs) via electrostatic interaction-induced self-assembly. The assembled Gd-PNPs exhibited excellent fluorescent imaging, with a significant increase in the fluorescence lifetime of Gd–TCPP likely due to the severely restricted molecular motion and substantially weakened stretching and bonding vibrations after encapsulation. The assembled Gd-PNPs also displayed high *r*_1_ relaxivity (16.16 mM^–1^ s^–1^ at 1.5 T) which is significantly higher than most commercially available T_1_-weighted MRI CAs. They also exhibited long-term colloidal stability, dispersity, and biocompatibility, along with enhanced in vivo fluorescence/MRI-guided tumor growth inhibition efficiency in CT 26 tumor-bearing mice. The enhanced MRI is likely due to the altered rotational behavior of the assembled composite, and also the high number of coordinated water molecules to this Gd-TCPP complex, although this is not thoroughly probed. Though useful, such systems are not ideal, unless their kinetic stability can be ascertained, due to the potential safety implications of Gd^3+^ leaching.

The self-assembly of polyelectrolytes through electrostatic interactions offers simplicity, flexibility, and versatility, making it an attractive approach for designing functional MRI CAs. However, electrostatic interactions are highly sensitive to ionic strength, which is a concern in biological environments like blood or intracellular fluids, where high ionic strength may weaken these interactions. This can lead to instability or disassembly of the polyelectrolyte complex, potentially reducing the performance, effectiveness, and ultimately the clinical utility of MRI CAs in vivo.

### 4.2 Self-Assembly of Amphiphilic Polymers

Among the diverse range of polymeric components useful in self-assembly processes, amphiphilic polymers, featuring both hydrophilic and hydrophobic segments, stand out as highly effective building blocks. In mixed phase systems, the aqueous-soluble hydrophilic portion interacts predominantly with the aqueous phase, while the hydrophobic segment tends to reside in the air or nonpolar solvent, leading to aggregation of the amphiphiles. This forms various macromolecular assemblies driven by the repelling and coordinating forces between the hydrophilic and hydrophobic components and the surrounding medium.^92,97–99^

Du et al. sought to exploit this in their preparation of polymer vesicles using two types of amphiphilic diblock polymers with an identical polymer structure but differing in poly­(l-glutamic acid) (PGA) chain length, namely R-poly­(l-glutamic acid)-*block*-poly­(ε-caprolactone) [R is folic acid (FA) or DTPA] (FA/DTPA-PGA-*b*-PCL) (Figure 1c).^74^ The combination of these polymers led to the self-assembly of asymmetrical vesicles with an average diameter of about 150 nm. The membrane of these vesicles consisted of biocompatible and biodegradable PCL. The outer corona of the polymer vesicle was designed using biodegradable hydrophilic PGA with a longer chain length and an FA terminal group (for cancer targeting), while the inner corona featured short PGA with a DTPA chelating agent, allowing Gd^3+^-chelation for MRI activity. This system resulted in an exceptionally high T_1_ relaxivity (*r*_1_ = 42.39 mM^–1^ s^–1^ at 37 °C at 1.41 T, 8-fold better than Gd^3+^-DTPA alone); although not specifically measured, this enhancement is likely primarily due to the increased τ_R_ as Gd^3+^ complexes are covalently conjugated onto polymeric assemblies, but the enhancement may also benefit from their confinement within a water pool inside the assembled vesicles. An additional feature of this system was its efficient anticancer drug loading within the hydrophilic cavity, where doxorubicin hydrochloride, DOX·HCl was loaded at a high efficiency of 52.6%. With the exploitation of a designed water pool, and controllable degradation, such systems also offer the opportunity for using changes in MRI signals to monitor the degradation, an emerging research trend of interest pharmaceutically.^102^

### 4.3 Peptide/Protein/DNA-Assisted Self-Assembly

There has been increasing interest in exploring peptide, protein, and DNA-based building blocks for the creation of self-assembled nanostructures due to their applications in the field of drug delivery, gene therapy, biosensing, imaging and diagnostics. Peptides, proteins, and DNA molecules assemble through a variety of forces and interactions, including hydrogen bonding, electrostatic interactions, hydrophobic interactions, and π-π stacking. From an MRI perspective, the signal enhancements are mechanistically similar to those described in section 3.1 (i.e., due to reduced global and local rotation rates). However, their versatility in assembly mechanisms distinguishes peptide/protein/DNA-assisted assembly from other polymeric CAs which exploit similar interactions.^103–107^ Three representative studies are explored here using the different material types, illustrating the diversity of approaches within this framework.

Along these lines, Accardo et al. prepared peptide materials based on the aggregation of polyphenylalanine conjugates containing gadolinium complexes, demonstrating potential as MRI CAs.^108^ The building block monomers featured two (F2) or four (F4) phenylalanine residues for self-assembly through the interactions between the phenylalanine side chains, along with a chelating agent (DOTA or DTPA) to achieve gadolinium incorporation and MRI activity. Ethoxylic linkers at two (L2) or six (L6) poly­(ethylene glycol) (PEG) units were incorporated between the chelating group and the peptide region. Both Gd-DOTA and DTPA tetraphenylalanine derivatives exhibited self-aggregation at very low concentrations, forming amyloid type fibril formation with stacking and π-π intermolecular interactions. The *r*_1_ relaxivities of the two complexes were 14.8 and 14.0 mM^–1^ s^–1^ for DOTA­(Gd)-L6-F4 and DTPA­(Gd)-L6-F4, respectively (0.5 T and 25 °C), far surpassing classical low-molecular weight CAs due to their overall increased τ_R_ rotational correlation time (verified using Nuclear Magnetic Relaxation Dispersion, NMRD). Interestingly here, the NMRD analysis shows faster than expected (for nanostructures) τ_R_ values, which is attributed to fast internal motility of the Gd-complexes due to their location on the linker. This detailed study illustrates the importance of in-depth analyses and mechanistic enhancements beyond changes in bulk alone.

Hamilton et al. reported the preparation of CAs based on gadolinium complexes conjugated to a self-assembling DNA quadruplex scaffold.^109^ This pioneering work found that for a single Gd-DOTA chelated DNA strand, the *r*_1_ was 6.4 mM^–1^ s^–1^, which increased to 11.7 mM^–1^ s^–1^ upon formation of a DNA quadruplex, despite its small size of only 2.5 nm when assembled. Similar results were observed when a Gd-DOTA dendrimer featuring several Gd-DOTA groups was conjugated to DNA, with the *r*_1_ increasing to 12.9 mM^–1^ s^–1^ upon the formation of a DNA quadruplex, compared to 6.0 mM^–1^ s^–1^ for a single strand. This equates to an *r*_1_ of 154.8 and 46.8 mM^–1^ s^–1^ per DNA quadruplex for the DOTA dendrimer and monomer, respectively (all relaxivities were measured at 4.0 T and 20.5 °C). Although not the traditional manner of reporting relaxivity values, this work shows that significant enhancement could be obtained with even small levels of uptake in the body. Interestingly here, the enhancement is likely to be a combination of increased rotational time due to the slightly larger quadruplex, together with different levels of linker flexibility which are offset by the ordered quadruplex scaffold.

The self-assembly of biomaterials, such as peptides, proteins, and DNA, as MRI CAs takes advantage of their inherent biological nature, which endows them with precise molecular recognition, biocompatibility, and responsiveness to biological stimuli.^110,111^ These properties enable selective interactions, reducing unintended effects and improving both diagnostic accuracy and therapeutic effectiveness in complex biochemical environments. Despite the promising potential, modularity, and versatility of these biomaterial-based strategies in creating well-defined nanoscale materials, their development is still in the early stages, and the structural stability in vivo needs exploring.

As discussed above, the enhanced performance of assembled small-molecule and polymeric T_1_ contrast agents, compared to their non-assembled counterparts, primarily arises from the formation of supramolecular aggregates with prolonged rotational correlation times (i.e., decreased tumbling rates). Notably, the tumbling dynamics are influenced not only by molecular weight but also by the geometry of nanoassemblies. For example, in a study conducted by Lee et al., a series of antimicrobial peptides conjugated with Gd^3+^-chelating moieties or cell-targeting moieties were prepared.^112^ These peptides could self-assemble into various structures, including micelles, fibrils, vesicles, sheets, and planar networks. The investigation revealed that, among all the assembled nanoconstructs, the sheet-like structure exhibited the highest *r*_1_ value. The authors propose that the self-assembled two-dimensional (2D) planar sheet configuration markedly prolongs the rotational correlation time compared to other nanostructures. However, the interplay between nanostructure morphology and variations in the molecular weight and chemistry of the constituent peptides introduces considerable complexity. Similar structure-dependent behaviors have been reported in other studies,^113–115^ highlighting how the shape and form of nanoassemblies can strongly influence MRI performance. Importantly, this structure-dependent effect is a unique feature of the assembled state rather than of the individual molecular or polymeric components. A comprehensive understanding of structure-dependent MRI behavior in assembled T_1_ contrast agents remains incomplete, and findings in this area are sometimes inconsistent. Therefore, further systematic investigations are needed to clarify the precise role of self-assembled construct features, and researchers should consider the potential structural outcomes of self-assembly when designing chelates and polymeric contrast agents intended for assembly.

## 5 Developing Strategies for the Self-Assembly of Inorganic MRI CAs

Inorganic nanoparticle assembly is a complex and versatile method for creating novel materials, with the capability to utilize thousands of different combinations of particle sizes, shapes, compositions, and ligand chemistries to produce a diverse array of structures. Depending on their final construct, the enhancement of MRI signal from such assemblies can result from anisotropic influences, changes in water accessibility to MRI active surfaces or components, or alterations in the water exchange rate. As such, assembly of inorganic particles offer the widest array of approaches and almost unlimited potential improvements in contrast signal, as long as the mechanisms of signal enhancement are well understood and executed. Examples in this section are selected to demonstrate themes of enhanced relaxivity due to magnetic interactions, and the continual improvements in these with tailored synthetic approaches.

### 5.1 Assembly Assisted by Geometrical Confinement

The ability to organize nanoparticles (NPs) into ordered structures, anticipating collective physical or chemical properties, has opened new opportunities in nanotechnology.^121–124^ While one-dimensional templates (for example anodic aluminum oxide)^118^ and two-dimensional interfaces (like liquid–liquid or liquid–air interfaces) serve as confined spaces for assembly,^119,120^ this perspective aims to concentrate on the three-dimensional (3D) spatial confined assembly of nanoparticles. This form of assembly is widely recognized as the most representative and commonly observed among nanoparticle structures and furthermore facilitates MRI improvements through harnessing well-established mechanisms of signal enhancement such as dipolar-mediated enhancements, increased magnetic field inhomogeneities, and intimate water-magnetic surface interactions.^41,98–100^ Approaches to yield confined magnetic systems are typically based upon emulsion-driven techniques, where Pickering emulsions are an important intermediate step, however assembly into structured spaces is becoming a more popular method of assembly, although it lacks the elegant precision of emulsion methods.

#### 5.1.1 Assembly within Microemulsions

Microemulsions are colloidal dispersions that are thermodynamically stable, characterized by the coexistence of two immiscible liquids, typically water and oil, within a single phase. This stability is achieved through the presence of a monolayer of (solid) surfactants with balanced hydrophilic–lipophilic properties.^125–127^ Microemulsions serve as a versatile platform for synthesizing high-quality inorganic nanoparticles^128^ and provide a facile method for preparing ordered assemblies, aiding in the analysis of structure-dependent MRI performance.^129^ An interesting approach to this was described by David et al., who prepared magnetic nanostructure-stabilized lipid nanocapsules (MLNCs) by combining citrate-stabilized, negatively charged magnetic nanoparticles Zn_0.2_Mn_0.8_Fe_2_O_4_ (in the aqueous phase) with cationic lipids in chloroform (in the oil phase).^130^ Through self-emulsification, oil-in-water Pickering emulsions were formed with lipid-magnetic nanoparticles situated at the liquid–liquid interface. After the evaporation of chloroform, nanocapsules with a hydrodynamic size of 130 nm were produced. The magnetic resonance contrast enhancement of the MLNCs (with a *r*_2_ relaxivity of 680 mM^–1^ s^–1^ at 3.0 T) is nine times higher than that of a clinically approved T_2_ MRI CA (ferumoxytol, 81 mM^–1^ s^–1^), demonstrating the superior diagnostic imaging capability of the MLNCs in MRI. This is because the nanoclusters of magnetic nanoparticles within the capsules generate a stronger magnetic moment per unit volume than isolated magnetic nanoparticles. On the other hand, the parent magnetic particles themselves exhibited reasonably high *r*_2_ of 425 mM^–1^ s^–1^, so the assembly induced enhancement likely could be optimized further. A beneficial feature of these MLNC-type constructs is their versatility – MRI signal can be augmented through tailoring of the composition and magnetic properties of the magnetic component. However, the case-specific key limitation for microemulsions is the potential toxicity of the surfactants required for their formation. The historical toxicity profile of many surfactants, due to their membrane-disrupting properties, presents a distinct hurdle that must be proactively addressed through the careful selection of biocompatible, or novel/green surfactants.^131–133^

Microfluidic techniques, as an advanced microemulsion-assisted assembly approach, hold significant promise for precisely controlling the assembly of micro- and nanoscale building blocks through the thoughtful design of various microfluidic environments.^134^ This approach represents an important variation to bulk microemulsions, since control over the assembled species influences neighboring dipolar interactions which can significantly impact the resulting relaxivities, and final emulsion sizes can also influence water interactions with magnetic surfaces.

Despite their popularity, and the use of magnetic components in these systems, their (typically) micrometer size dimensions hinder their application in MRI. One recent study which reduces particle sizes to MRI-relevant dimensions and relates the structure-MRI-property relationships of confined particles is by Gao et al., who utilized double emulsions generated through the combined effect of self-emulsification and phase separation of a water-dichloromethane-hydrophobic iron oxide system to confine the self-assembly of magnetic nanoparticles (MNPs), resulting in the preparation of hierarchically structured magnetic single-hole hollow spheres (MSHS) (Figure 2a,b).^116^ The MSHS exhibited an overall size of ∼650 nm, with hole diameters ranging from 150 to 400 nm. The saturation magnetization (M_S_) values for MSHS-150, 300, and 400 (the difference here is the diameter of the holes) were determined to be 76.7, 65.2, and 60.5 emu/g, respectively, nearly twice as high as that of individual Fe_3_O_4_ nanoparticles. MSHS demonstrated significantly higher *r*_2_ values (56, 662, 681, and 694 mM^–1^ s^–1^ for individual nanoparticles, MSHS-150, 300, and 400, respectively), surpassing those of the commercially used Feridex (98.3 mM^–1^ s^–1^ at 3.0 T) by more than 6-fold. The authors proposed that the significant relaxivity enhancement compared to the individual particles is likely due to the stronger magnetic dipole–dipole interactions between MNPs in a well-organized assembly, which are more pronounced compared to interactions among individual nanoparticles or randomly aggregated patterns. This correlates with earlier studies describing clustered particles.^135^ The stronger T_2_ effect observed in MSHS-400, despite its relatively lower *M*_S_ compared to MSHS with smaller holes, could be due to the synergistic influence of larger pores and increased surface hydrophilicity, which together improve water accessibility. There may be some limitation in practical application due to the large size of these particles, making smaller analogues desirable. Addressing this issue, in another study, Biswas et al. developed the rapid synthesis of lipid/iron oxide hybrid nanoparticles with a diameter of 50 nm and very similar features of magnetic particles assembled at a lipid particle surface.^136^ The successful reduction in particle size achieved through microfluidics-based synthesis represents a notable advancement, markedly enhancing the potential application of these assembled iron oxide hybrid nanoparticles as MRI CAs.

**Figure 2:**
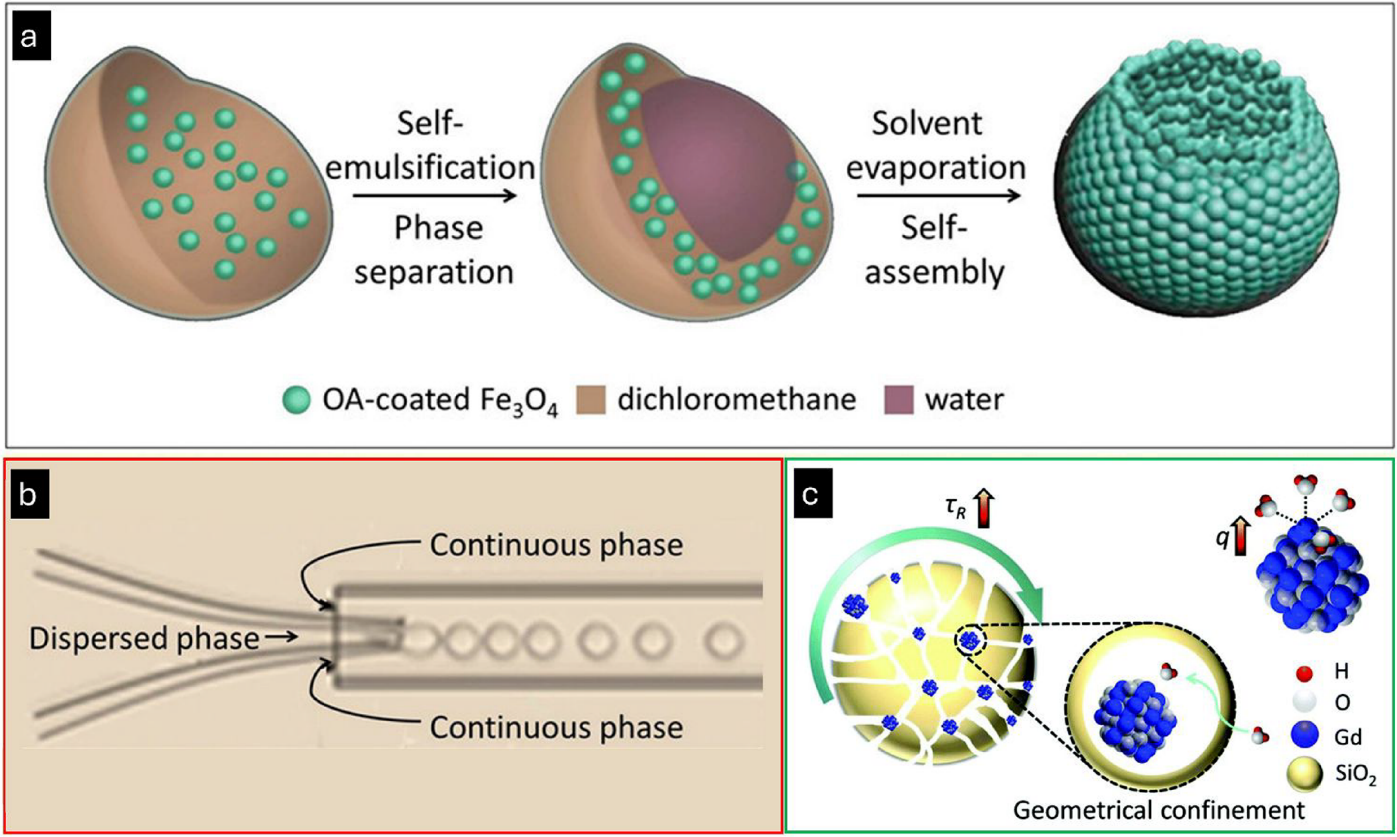
(a) Schematic illustration of the self-assembly strategy based on double emulsions to produce magnetic single-hole hollow spheres (MSHS). (b) Formation of oil-in-water droplets using a microfluidic device and the corresponding optical micrographs of single droplets and double emulsions. The dispersed phase consists of magnetic nanoparticles and polyethylene glycol in dichloromethane, and the continuous phase is the aqueous phase of sodium dodecyl sulfate solution. Adapted with permission from ref 116, copy right 2016 American Chemical Society. (c) Schematic illustration of the composite material with ultrasmall Gd_2_O_3_ nanoparticles immobilized inside worm-like interior channels of mesoporous silica nanoparticles (Gd_2_O_3_@MSN), highlighting strong water interactions responsible for high relaxivities. Adapted with permission from ref 117, copy right 2016 Royal Society of Chemistry.

#### 5.1.2 Assembly in Porous Materials

Ordered porous materials, such as MOFs,^137^ porous carbon,^138^ and hierarchical zeolites,^139^ provide a unique platform for the self-assembly of inorganic nanoparticles. Their uniform pore sizes, high pore volumes, and tunable surface chemistries enable controlled spatial confinement, influencing the physical and chemical properties of the assembled nanoparticles. Among these materials, mesoporous silica nanoparticles (MSNs) stand out as a representative system due to their well-established synthesis, precisely controlled pore structures, high surface area, good aqueous stability, and excellent biocompatibility. These properties make MSNs particularly attractive for geometrically confined clustering, where assembled nanoparticles can exhibit increased magnetic susceptibility, higher magnetization, and consequently enhanced relaxation effects. A neat example of this was described by Deb et al., who employed a solvent evaporation-driven assembly strategy to load magnetic nanoparticles into a mesoporous framework to design a highly efficient MRI contrast probe.^140^ Hydrophobic iron oxide nanoparticles were absorbed into the pores of MSNs (in the presence of an aqueous cetyltrimethylammonium bromide emulsion) due to the continuous shrinkage of the hydrophobic environment under evaporation conditions. This led to clusters of iron oxide particles residing inside the porous MSN networks, with reported transverse relaxivity (*r*_2_ measured at 9.4 T) increasing to 386.2 mM^–1^ s^–1^ for the internally assembled nanoparticles (from 191.8 mM^–1^ s^–1^ for individual nanoparticles), and *r*_2_/*r*_1_ ratios of 49.20 and 9.59, respectively. The authors suggested that the improved performance of the assembled particles was due to the high concentration of nanoparticles in the radial pores, where water protons are strongly influenced by varying magnetic fields from the embedded particles, contrary to individual suspensions of particles. It is also likely that altered water mobility and diffusion coefficients in these confined spaces influence the water accessibility and exchange properties, although this verification is not provided.^141–143^

In the preparation of high-performance T_1_ CAs, ultrasmall gadolinium oxide (Gd_2_O_3_) nanoparticles could also be geometrically confined within MSN channels, as demonstrated by Gao et al. (Figure 2c).^117^ *r*_1_ values of Gd_2_O_3_ and geometrically confined Gd_2_O_3_ nanocomposites measured at 3.0 T were 11.19 mM^–1^ s^–1^ and 16.95 mM^–1^ s^–1^, respectively. The unique Gd_2_O_3_@MSN structure endowed the Gd_2_O_3_ nanoparticles with geometrical confinement, increased molecular tumbling time, and a high number of coordinated water molecules, resulting in a significant enhancement of T_1_ contrast. Despite this enhancement, caution with such species is vital, since leaching of Gd^3+^ can occur, with potential safety risks.

Compared to microemulsions and microfluidic strategies, assembling within porous materials can be more complex, requiring precise control over synthesis conditions to achieve uniform distribution and optimal interaction between components. Since the relaxation enhancement is influenced by a number of parameters, this complexity may affect the consistency and effectiveness of the MRI CAs, as uneven particle distribution or inadequate interaction between the iron oxide nanoparticles and the porous matrix could limit the contrast enhancement and overall imaging performance.

### 5.2 Polymer-Mediated Assembly

Polymer-functionalized inorganic nanomaterials, combining the versatile properties of polymers and inorganic nanomaterials, have garnered significant attention across various fields. In the context of inorganic nanoparticle MRI CAs, various types of polymers have been used to assemble with inorganic MRI CAs to achieve well-designed functional composite materials.^144^ Signal enhancements by this approach again exploit clustering-mediated improvements, with the hydrophilicity of many polymers and their hydration networks also aiding in boosting MRI contrast.

#### 5.2.1 Amphiphilic Polymer-Assisted Assembly

The combination of superstructure-directing amphiphilic block copolymers with inorganic nanoparticles represents the predominant method for polymer-mediated assembly, leading to the formation of nano/microcomposites with obvious advantages in MRI due to their close particle interactions. For example, Nie et al. developed magneto-vesicles (MVs) using the assembly of polystyrene-*b*-poly­(ethylene oxide) (PS-*b*- PEO)-tethered SPIONs and free poly­(styrene)-*block*-poly­(acrylic acid) (PS-*b*-PAA).^146^ The thickness of MV membranes, ranging from 9.8 to 93.2 nm, was tunable by adjusting the weight ratio of PS-*b*-PAA to SPIONs, leading to the formation of monolayered MVs (MoMVs), double-layered MVs (DoMVs), and multilayered MVs (MuMVs). Thicker membrane MVs exhibited higher magnetization, enhancing magnetic manipulation, and increased *r*_2_ MRI relaxivities. The *r*_2_ value measured at 7.0 T for MuMVs was 293.6 mM^–1^ s^–1^, higher than that measured for DoMVs (167.1 mM^–1^ s^–1^), MoMVs (149.9 mM^–1^ s^–1^), and individual SPIONs (108.7 mM^–1^ s^–1^). The increased *r*_2_ in MuMVs was attributed to the higher SPION density in vesicular membranes, enhancing overall magnetic moment and magnetization. Additionally, the MVs allowed efficient encapsulation and tunable release of therapeutic agents like doxorubicin.

#### 5.2.2 Peptide/Protein/DNA-Assisted Assembly

Biomolecules serve as intricate ligands for nanoparticle assembly, thanks to their macromolecular structures that facilitate sophisticated interactions. These interactions can be considerably more complex compared to those mediated by small-molecule ligands or synthetic polymers and therefore hold potential in directing nanoparticle and water interactions for favorable MRI enhancement.^147^ Furthermore, they permit development of switchable contrast signal, through modulated assembly/disassembly. An interesting and elegant example of a contrast switchable system using peptides was described by Song et al., who introduced a peptide-modified magnetic resonance tuning (MRET) probe, MPD-1, designed to detect bacterial infections in a mouse myositis model by responding to matrix metallopeptidase 2 (MMP-2) (Figure 3).^145^ MPD-1 is composed of a magnetic iron oxide (Fe_3_O_4_) nanoparticle (MNP, a T_2_ CA) conjugated with gadolinium ions (acting as a T_1_ CA), with key components including a MMP-2-cleavable self-assembled peptide (P1) and a bacteria-targeting peptide (P). Notably, the self-assembling ability of the “phenylalanine-phenylalanine” dipeptide in P1 allows the MPD-1 monomer to self-assemble through hydrogen bonding, π–π interactions, and hydrophobic interactions. This self-assembly brings the Gd^3+^ enhancer close to the MNP which acts as a signal quencher (MRET “ON”), resulting in a low T_1_ MRI signal for MPD-1. Upon accumulation at a bacterial infection site, P1 within MPD-1 is selectively cleaved by MMP-2, causing the disassembly of the T_2_ CA, leading to T_1_-weighted signal recovery due to removal of the quenching partner proximity (MRET “OFF”). Following MMP-2 cleavage, MPD-1 exhibited a substantial decrease in the *r*_2_/*r*_1_ ratio from 46.14 to 2.33, indicating the transformation of MPD-1 from a T_2_ to a T_1_ CA in response to the bacterial infection (measurements carried out at 0.5 T).

**Figure 3:**
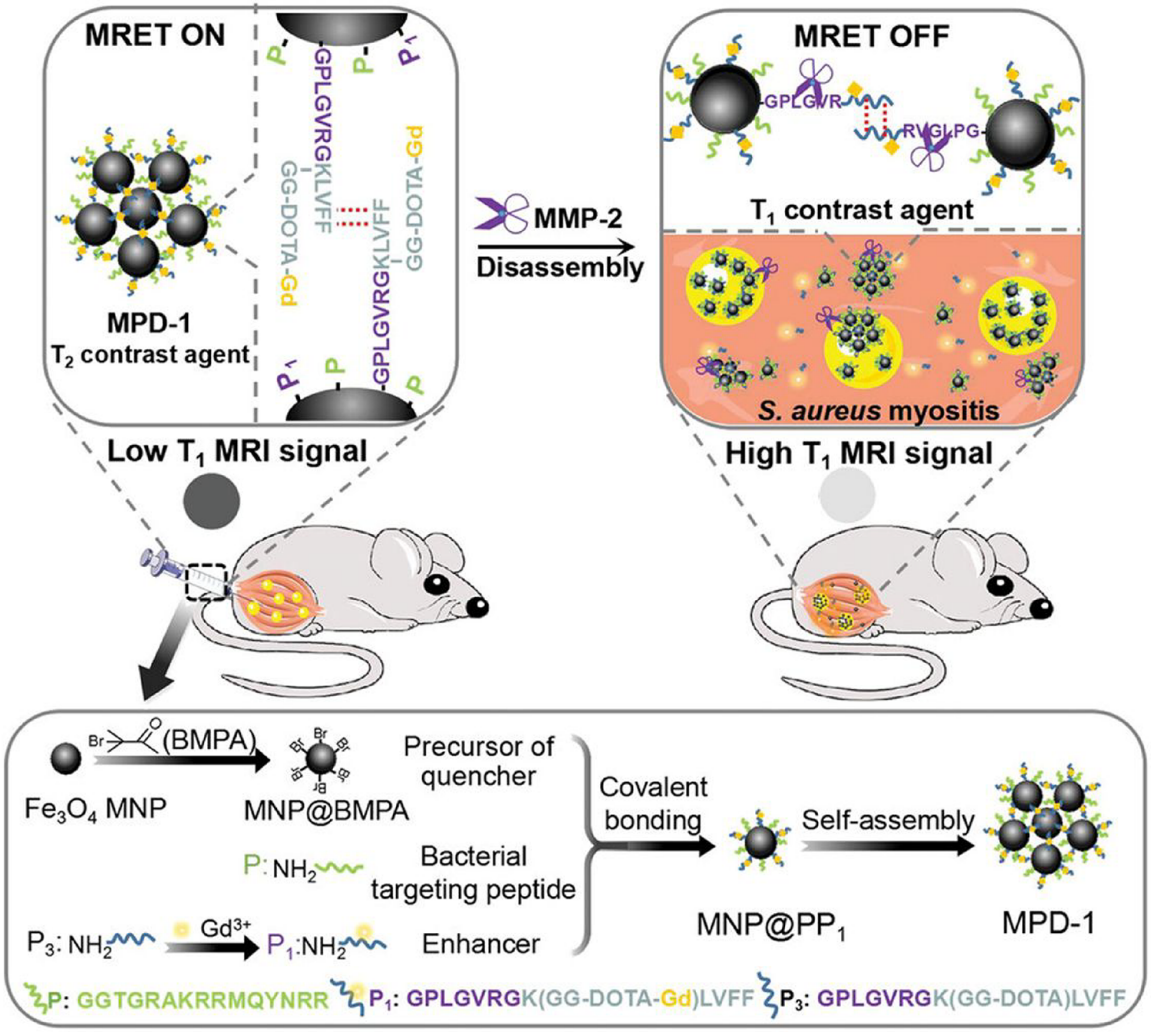
Schematic illustration of peptide-modified magnetic resonance tuning (MRET) probe (MPD-1) for in situ turn-on MRI of bacterial infection in vivo. Adapted with permission from ref 145, copy right 2023 John Wiley and Sons.

Ke et al. utilized a DNA origami rod to precisely manipulate the number and placement of iron oxide nanoparticles (IONPs) in an anisotropic assembly, showcasing the tunability of MRI contrast generation efficiency by adjusting the number and spacing of IONPs (and hence their magnetic dipolar interactions).^148^ The DNA origami rod, named 16 Helix Bundle (16HB), featured 16 double helices arranged in a 4 × 4 square lattice, with dimensions ∼10 nm × 10 nm × 130 nm. As the number of IONPs increased from 2 to 8, the *r*_2_ amplification (measured at 0.55 T, 30 °C) also increased from 110 to 139%. Generally, larger clusters significantly enhance *r*_2_ because water protons spend more time diffusing through the larger magnetic field inhomogeneities produced by the IONP clusters, leading to increased dephasing and higher relaxation rates. However, this research demonstrated that the enhancement from assemblies with fully saturated binding (approximately 8 IONPs/16HB) and close packing has an upper limit in *r*_2_. The relationship between the number of IONPs (N) and *r*_2_ follows a power law, resulting in a 250% increase in *r*_2_ when N reaches 8. This example correlates well with theoretical predictions and neatly illustrated the functional impact of highly controlled IONP assembly on MRI relaxivities.

#### 5.2.3 Intermolecular Interaction-Assisted Assembly

Reversible non-covalent interactions, including hydrogen bonding, π-π stacking, hydrophobic interactions, and host–guest binding, serve as effective mechanisms for driving the assembly of ordered nanoparticle materials with concurrent ability to control MRI signaling. Although these interactions are weaker compared to permanent covalent bonds, their transient nature allows particles to reorganize during the assembly process, thus preventing kinetic traps.^149^ This was demonstrated in a study conducted by Park et al., who immobilized dopamine hydrochloride-functionalized beta-cyclodextrin (β-CD) on the surfaces of SPIONs (SPIO@CD).^150^ Arginine-glycine-aspartic acid peptide-conjugated poly­(ethylene glycol) and paclitaxel (PTX) were then hosted in the β-CD cavity through high-affinity complexation. The resulting self-assembled magnetic nanoclusters not only demonstrated controllable PTX drug release and enhanced particle uptake in cancer cells but also improved negative MR contrast performance. The *r*_2_ relaxivity of SPIO@CD at 1.5 T was 161.4 mM^–1^ s^–1^ whereas the *r*_2_ of the self-assembled magnetic nanoclusters increased up to 261.6 mM^–1^ s^–1^, with this significant enhancement attributed to the increased magnetic moment from the cluster size and density.

Electrostatic interactions can maintain colloidal stability when all particles bear like charges, while ordered patterns can be achieved by utilizing sets of nanoparticles functionalized with oppositely charged ligands. This intriguing effect was illustrated to benefit MRI signal enhancement by Berret et al., who employed polyelectrolyte-neutral block copolymers to form stable clusters with oppositely charged iron oxide nanoparticles through electrostatic interactions.^151^ The copolymers were poly­(trimethylammonium ethyl acrylate methyl sulfate)-*b*-poly­(acrylamide), with molecular weights of 5000-*b*-30,000 g mol^–1^ and 110,000-*b*-30,000 g mol^–1^, and the negatively charged sister particles were citrate-stabilized IONPs. The size of the assembled networks could be adjusted by varying the ratio between the two oppositely charged components. *r*_2_ relaxivities (at 0.47 T, 37 °C) ranged from 39 ± 2 mM^–1^ s^–1^ for IONPs alone to 74 ± 4 mM^–1^ s^–1^ for clusters prepared with the 5–30K copolymer, peaking at 162 ± 4 mM^–1^ s^–1^ for clusters made with the 11–30K chains. This enhancement can be linked to the rotational behavior of the systems, evidenced by increasing hydrodynamic size, as well as inevitable magnetic NP dipolar interaction effects.

Compared to these non-covalent examples, nanoparticle assemblies covalently linked via rapid chemical reactions like “click” chemistry, serve as robust methods to fortify the assembled structure significantly. This reinforcement results in the creation of highly stable and enduring architectures, essential for maintaining structural integrity over time without aging or decay.^152^ This approach was illustrated by Long et al., who designed two sets of iron oxide nanoparticles with azide or alkyne groups concealed by PEG-linked tumor-targeting peptides that bind to the CXCR4 receptor.^153^ In the tumor microenvironment, matrix metalloproteinases (MMPs) specifically cleave the peptide linker at the base of the PEG moiety, exposing the copper-free cross-reactive azide and alkyne groups. This covalent reaction induces self-assembly of clusters, enhancing the MRI signal by increasing magnetic susceptibility when aggregates are formed, which leads to an enhancement in *r*_2_ relaxivity. In vitro testing revealed T_2_ signal enhancements of approximately 160% (at room temperature and 9.4 T) when the nanoparticles were incubated with cells expressing MMP2/9 and CXCR4.

Among the various approaches for assembling inorganic nanoparticles as MRI CAs, polymer-assisted methods are particularly attractive due to the complex architectures and diverse functionalities they enable. Despite significant advancements in this area, several challenges remain that require ongoing research. First, safety concerns related to polymers must be carefully addressed. Most importantly, to develop a predictive framework for controlling the self-assembly of desired hierarchical structures, a deeper understanding of the coassembly mechanisms across relevant length scales is essential. Achieving precise control over complementary interactions at the nanoscale remains a key challenge.

## 6 In Vivo Self-Assembly

In the previous sections, we explored pre-assembly before assessing the assembled CAs in in vitro or in vivo scenarios. Yet, in nature, the emergence of functional structures through the assembly of smaller units is a common occurrence within biological environments. Mimicking these naturally occurring structures by integrating synthetic self-assembled nanostructures into a biological context stands as a noteworthy advancement in supramolecular chemistry. By incorporating bioresponsive handles into the building block precursors, spontaneous postassembly within a biological environment can be achieved as the precursors are modified into stimuli-responsive materials responding to specific nanoenvironments or microenvironments within cells.^156^ Targeted in vivo biological stimuli, such as pH, redox reactions, or enzymatic activities, can be leveraged, with each stimulus often associated with a specific physiological environment.^157–159^ This approach allows for the preparation of self-assembled nanoprobes with enhanced sensitivity and specificity for MRI, which will only become active at the desired site.^93,159^

One representative example developed by Ye et al. described P-CyFF-Gd, a small-molecule-based bimodal imaging probe, for in vivo imaging. It utilizes an enzyme-triggered fluorogenic reaction and a subsequent in situ self-assembly strategy (Figure 4a).^154^ The probe consists of a prequenched NIR fluorophore (merocyanine, Cy-Cl) with an alkaline phosphatase (ALP) recognition phosphate group (−PO_3_H), a paramagnetic Gd-DOTA chelate for T_1_-MRI, and a hydrophobic dipeptide Phe-Phe (FF) linker to promote self-assembly. Initially water-soluble molecules with “off” NIR fluorescence and low *r*_1_ relaxivity (in their pre-assembly state), P-CyFF-Gd becomes activated by membrane-bound ALPs in ALP-positive tumor tissues. The released dephosphorylated product, CyFF-Gd, promotes molecular self-assembly, resulting in membrane-localized assembled nanoparticles visualized by cryo-SEM. The activated nanoparticle-based probe shows simultaneous enhancements in NIR fluorescence (>70 fold at 710 nm) and *r*_1_ relaxivity at 0.5 T (∼2.3-fold) due to the assembly, enabling real-time, high-sensitivity, high-spatial-resolution imaging of ALP activity in live tumor cells and mice.

**Figure 4:**
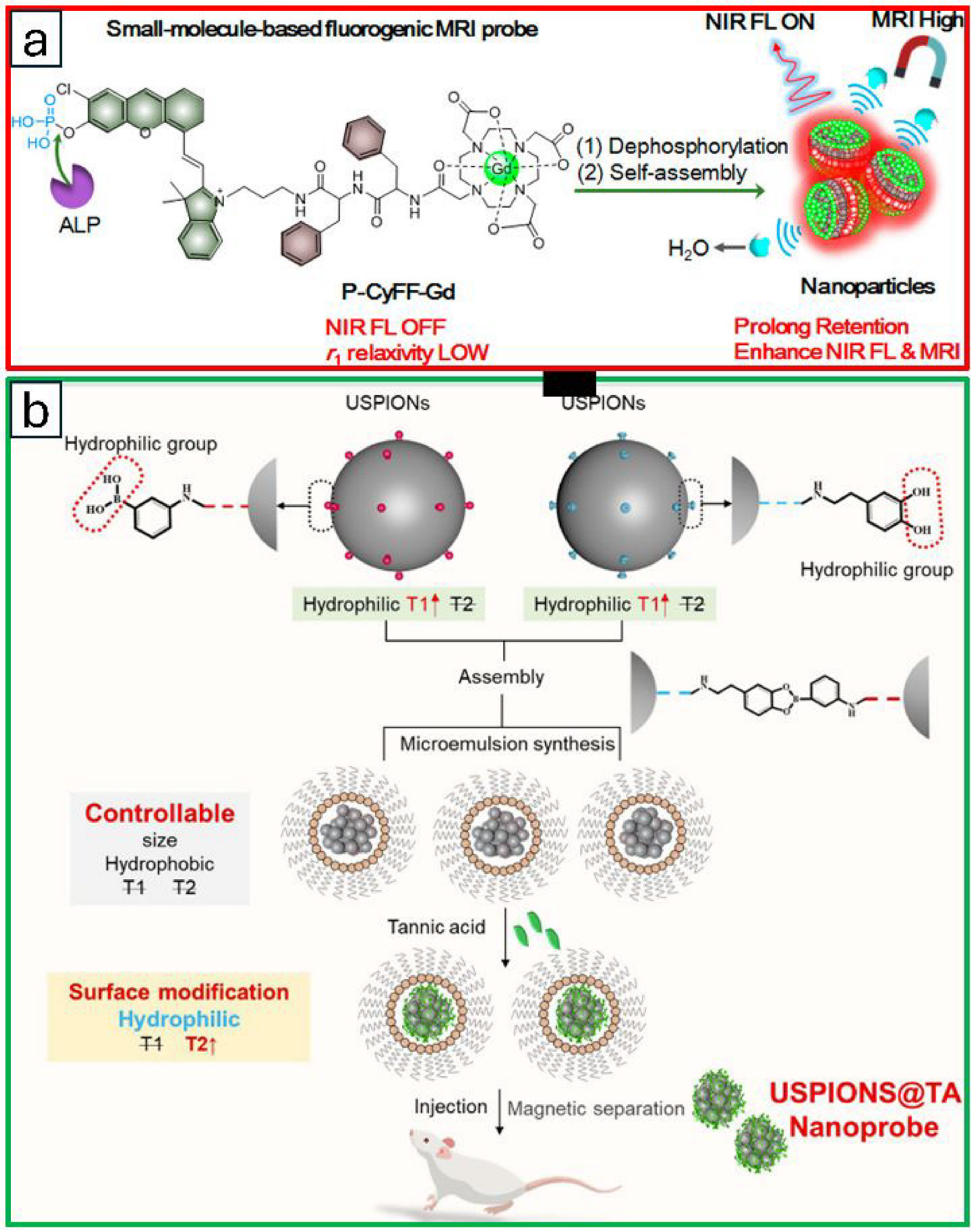
(a) Schematic illustration shows an alkaline phosphatase (ALP)-activatable near-infrared (NIR) fluorescence (FL)/MR bimodal probe for in vivo imaging. Adapted with permission from ref 154, copy right 2019 American Chemical Society. (b) Schematic diagram of the synthesis and sensing principle of ultrasmall SPIONs@tannic acid (USPIONs@TA) nanoprobes. Adapted with permission from ref 155, copy right 2022 American Chemical Society.

In a similar vein, Gao et al. introduced tumor microenvironment-responsive nanoprobes designed for improved tumor imaging using magnetic nanoparticles as building blocks.^160^ These nanoprobes consisted of Fe_3_O_4_ nanoparticles with a responsive peptide sequence, comprised of a tumor-specific Arg-Gly-Asp peptide for targeted tumor binding and a self-peptide serving as a “mark of self,” connected by a disulfide bond. Upon cleavage of the self-peptide by glutathione (GSH) within the tumor microenvironment, the exposed thiol groups reacted with the remaining maleimide moieties from adjacent particles, resulting in in situ cross-linking of the Fe_3_O_4_ particles. Experimental results from both in vitro and in vivo studies indicated that this aggregation significantly enhanced the MRI contrast performance of Fe_3_O_4_ particles. The T_2_ effect of the peptide-coated Fe_3_O_4_ particles showed a significant enhancement due to aggregation over time, leading to a rapid increase in the transverse relaxation rate *r*_2_ at 1.5 T by more than 1.82 during a 6-h incubation period. In contrast, the control probe exhibited nearly unchanged *r*_2_ over the same period of incubation with GSH.

Recent advances have demonstrated that self-assembly mechanisms can improve the ability to detect subtle changes in the cellular microenvironment. For instance, Li et al. reported α-synuclein oligomer-driven T_1_–T_2_ switchable nanoprobes (ASOSN) for early Parkinson’s disease diagnosis.^161^ These iron oxide nanoparticles, functionalized with single-chain fragment variable (scFv) antibodies, interact with hydrophobic regions of α-synuclein oligomers, enabling specific recognition. In α-synuclein–expressing cells, ASOSN gradually assembled over time, producing switchable MRI signals. In contrast, BV2 cells lacking oligomers displayed only a reduced T_1_-weighted signal without changes in T_2_ contrast. Relaxometric analysis at 9 T confirmed ASOSN’s dual-mode behavior: initially, ASOSN exhibited *r*_2_/*r*_1_ ≈ 1.77, characteristic of a T_1_ agent. Upon oligomer-induced assembly, the *r*_2_/*r*_1_ ratio increased dramatically to ≈ 36.5, consistent with strong T_2_ contrast.

Compared to ex situ methods, in situ self-assembly strategies triggered by intelligent stimuli using (macro-)­molecules to form nanoparticles at targeted sites offer enhanced signals and prolonged retention, making them more efficient for imaging. However, there is a critical need for new, convenient, and biocompatible systems or reactions to intracellularly assemble nanostructures for both imaging and therapeutic applications, enabling more specific, efficient and sensitive imaging and imaging-guided therapy.

## 7 Assemblies for T_1_-T_2_ Dual or Switchable MRI CAs

Self-assembly of CAs not only leads to improved T_1_ or T_2_ nanoaggregates, but also provides an avenue for T_1_-T_2_ dual CAs, as well as T_2_-T_1_ switchable CAs. The development of dual-mode CAs, capable of facilitating both T_1_ and T_2_ imaging modes simultaneously, represents a significant advancement in MRI. These agents offer the advantage of providing complementary diagnostic information within a single imaging session, enhancing diagnostic accuracy and efficiency.^162^ The flexibility to choose between T_1_ and T_2_ modes allows for tailored imaging based on specific diagnostic needs. Wang et al. presented one of the first proof-of-concept studies of this involving hybrid nanogel-based MRI CAs serving as T_1_-T_2_ dual-mode MRI CAs.^163^ They utilized acryl bonds and biotin group-functionalized glycol chitosan as polymeric ligands to assemble SPIONs. To enhance interaction between T_2_ and T_1_ species, potassium permanganate was subsequently in situ reduced to Mn oxides (MnO_*x*_) on starch-*g*-poly­(acrylic acid-*co*-biotin) (producing SPIO@GCS/acryl/biotin@Mn). The resulting SPIO/Mn/hydrogel composites exhibited low *r*_1_ and *r*_2_ behavior due to mutual quenching effects, however they were highly efficient for pH-responsive T_1_ (from MnO_*x*_) and T_2_ (from SPIONs) dual-modal MRI. The authors suggested that the in situ reduction of Mn species within the SPIO clusters disrupted their magnetization, causing a significant drop in saturation magnetization from 46.0 to 32.9 emu/g (and concurrent low *r*_2_). At the same time, when the newly formed MnO_*x*_ directly contacted the Fe_3_O_4_ nanoparticles, the magnetic field generated by the superparamagnetic T_2_ contrast material interferes with the relaxation process of the paramagnetic T_1_ contrast material.^164^ In in vitro tests, the dual-mode nanoassemblies demonstrated pH-responsiveness, with both T_1_ and T_2_ relaxivities turning “ON” in acidic environments, with the enhanced T_1_ effect observed attributed to the release of Mn ions. This resulted in a 1.7-fold increase in the *r*_1_ relaxivities (from 8.9 to 15.3 mM^–1^ s^–1^) and a 4.9-fold increase in *r*_2_ (from 45.7 to 226 mM^–1^ s^–1^), respectively (at 3.0 T), due to desirable desilencing (recovery of the saturation magnetization) effects. T_1_–T_2_ dual-modal MRI CAs improve diagnostic accuracy by providing complementary anatomical images with distinct contrasts, enhancing detection sensitivity and reducing artifacts for more reliable results. However, precise control of the distance between paramagnetic and superparamagnetic moieties is crucial to prevent T_1_ signal quenching due to the distance-dependent magnetic resonance tuning effect.^165,166^

Another highly intriguing direction for dual-modality systems is the development of dynamically switchable MRI CAs capable of regulating the activation and deactivation of imaging signals in both diseased and normal tissues. The principle is that upon arrival at the diseased site, these dynamic agents exhibit responsiveness to stimuli within the diseased microenvironment, eliciting structural alterations. Consequently, these modifications impact water proton relaxation dynamics, thereby facilitating the activation and switching of T_1_ and/or T_2_ MRI signals, which can then be monitored non-invasively through imaging.^167^ This concept has recently gained much attention, with a number of groups preparing ultrasmall SPIONs (USPIONs) based T_2_-T_1_ switching MRI CAs.^168–174^ A recent example provided by Li et al. involves the preparation of 3 nm USPIONs as T_1_ CAs, functionalized with dopamine (DM) and 3-aminophenylboronic acid (APBA) (Figure 4b).^155^ Through the boronic acid of APBA and the catechol groups of DM, the particle mixture became hydrophobic. The particles were then modified with tannic acid (USPIONs-TA), leading to the formation of assembled aggregates exhibiting enhanced T_2_ MRI signals (and suppressed T_1_ signal). This is because when the USPION nanoparticles aggregate, the clusters create stronger and more inhomogeneous local magnetic fields, hence amplifying dephasing effects on nearby water protons, thereby enhancing the T_2_ contrast effect.^15^ Interestingly, in this study, the nanoaggregates could undergo disassembly in the presence of acid and H_2_O_2_, allowing the discrete USPIONs to regain their T_1_ contrast ability. At pH 7.4, the relaxivity values (*r*_1_ and *r*_2_) of USPIONs@TA nanoprobes in aggregated state were 3.96 and 351.57 mM^–1^ s^–1^, respectively, whereas a lower pH buffer (pH 6.8) containing 100 μM H_2_O_2_ led to the disassembly and their corresponding values changing to 16.67 and 231.05 mM^–1^ s^–1^, as measured using a 1.5 T MRI instrument. Smart dynamic T_2_-T_1_ switchable MRI CAs hold great potential for early and accurate disease diagnosis but remain in the proof-of-concept stage.^175^ Their success depends on overcoming challenges such as ensuring high-sensitivity and selectivity to detect subtle microenvironmental changes. Optimizing stimuli-responsive ligands for better stability, specificity, and responsiveness is essential to achieve sensitive MRI activation at disease sites.

In vivo self-assembly has been exploited to enhance MRI performance by leveraging the responsiveness of contrast agents to the local cellular microenvironment, including pH, enzymes, and reactive oxygen species. This approach has also been applied to bioresponsive T_1_–T_2_ dual or T_2_–T_1_ reversible contrast agents, which can switch contrast modes in response to environmental cues. Signaling pathways in the human body are essential for maintaining organ function and are closely linked to disease development. Bioactive ions, such as Ca^2+^, K^+^, and Na^+^, as well as bioactive molecules, including neurotransmitters and gaseous signaling molecules (e.g., nitric oxide, carbon monoxide, and hydrogen sulfide), regulate these pathways through interactions with their receptors.^176,177^ Bioactive ion- and molecule-functionalized CAs hold great potential as smart nanosensors for receptor imaging, which is particularly valuable to provide critical insight into organ health and disease.^178–185^ For example, calcium ions (Ca^2+^) are universal signaling molecules, but current optical methods cannot monitor calcium dynamics deep in intact tissues. To address this, Jasanoff et al. reported the development of calcium-responsive magnetic nanoparticles (MaCaReNas) for MRI detection of extracellular calcium signaling.^179^ MaCaReNas were constructed by combining lipid-coated iron oxide nanoparticles (LCIOs) with the calcium-binding C2AB domains of synaptotagmin-1. In response to Ca^2+^ (0.1–1.0 mM), C2AB-mediated assembly increased hydrodynamic diameter from 35 ± 1 nm to 262 ± 14 nm and produced strong MRI contrast changes. The transverse relaxivity (*r*_2_) at 7 T rose from 151 ± 15 to 261 ± 21 mM^–1^ s^–1^ with half-maximal response at 0.43 mM Ca^2+^, while Mg^2+^ and control variants lacking calcium-binding activity showed no effect. Importantly, MaCaReNas enabled repeated in vivo detection of brain activation in response to diverse stimuli, establishing them as a powerful tool for deep-tissue calcium activity mapping by MRI. Although signaling ion- and molecule-responsive self-assembling MRI probes are still at an early stage and face challenges in biocompatibility, assembly stability, and signal specificity under physiological conditions, ongoing advances in contrast agent design and the integration of responsive elements are expected to accelerate their clinical translation, ultimately enabling earlier and more accurate detection of disease-related signaling changes.

## 8 Outlook

MRI stands out as a pivotal non-invasive biomedical imaging technique, renowned for its exceptional spatial resolution, remarkable soft tissue contrast, and profound tissue penetration capabilities. Extensive research efforts have been directed toward enhancing the contrast between pathological and normal tissues through the use of CAs. These CAs can be categorized based on their components into small-molecule, polymeric, and inorganic nanoparticles, or based on their site of action into extracellular, organ-specific, and blood pool agents. Optimizing the chemical structure of CAs is an essential approach toward improving their MRI performance. Theoretical relaxivity maxima for small-molecule chelates, such as Gd^3+^ and Mn^2+^, provide critical benchmarks for design,^186^ yet these values remain largely aspirational due to practical limitations in water exchange kinetics, rotational dynamics, and biocompatibility.^187^ Recent advances in ligand architecture demonstrate progress toward closing this gap; for example, Botta and colleagues engineered α-aryl substituted Gd-DOTA chelates with relaxivities of 11.7 mM^–1^ s^–1^ at 1.5 T, 2–3 times greater than conventional agents, with values increasing to 110 ± 5 mM^–1^ s^–1^ upon binding to human serum albumin, nearing theoretical limits for optimized molecular and protein-bound systems.^64,188^ Assessment of new CAs against theoretical maxima (at relevant field strengths, solvents, and conditions) is important; however, caution must be exercised when evaluating and comparing these limits, as emerging findings in the literature reveal relaxivity enhancements that are not fully explained by established theory.^189^ For example, water sequestration due to unique nanoparticle surface curvature of Au-nanostars appended with Gd^3+^-chelates was associated with significant *r*_1_ enhancements (54.7 mM^–1^ s^–1^ at 60 MHz and at 37 °C), not directly accounted for within traditional or refined SBM equations.^190^ Beyond small-molecule systems, inorganic contrast agents rely on distinct parameters, such as size, shape and surface chemistry, with optimization of nanoparticle design critical to enhancing relaxometric performance. While various theoretical frameworks define upper bounds of achievable relaxivity in such systems, intricate particle engineering can result in deviations from models.^64^ For instance, octapod-shaped iron oxide particles (30 nm edge length) exhibit an exceptionally high transverse relaxivity (*r*_2_) of 679.25 ± 30 mM^–1^ s^–1^ at 7 T, a 5.4-fold improvement over spherical equivalents; behavior attributed to an increased effective radius.^191^ Notably, these examples are not intended for direct comparison of *r*_1_ and *r*_2_ agents due to their different relaxation mechanisms, but to illustrate how construct design can impact relaxivity through distinct mechanisms beyond theoretical models. Crucially, some polymeric and inorganic agents often lack universally accepted theoretical maxima, due to complex properties (e.g., clustering and unique shape-dependent and anisotropic magnetization domains), requiring evolving theories. This emphasizes the need to evaluate performance through mechanisms unique to each class.

Meanwhile, recent strides in the field have been inspired by significant advancements in self-assembly methods and materials, with self-assembled CAs showcasing superior MRI performance compared to individual agents and exhibiting promising potential for switchable and active in vivo behavior, of value in clinical diagnostics. Despite the two distinct classes of positive (T_1_) and negative (T_2_) CAs being defined by different relaxation mechanisms, the features of each class have evolved to use similar design principles – self-assembly using electrostatic bonding, polymer and amphiphile assembly, among others, to guide the structure–property relationships and modulate MRI signal. Self-assembled CAs not only demonstrate considerably slower tumbling rates and enhanced water and active-species interactions (than non-assembled counterparts), but also generally manifest improved biodistribution, effective passive targeting to tumor sites, and tuneable clearance pathways when utilized as delivery carriers. However, it is important to avoid drawing direct parallels between the distinct relaxation mechanisms that may be involved.

New agents may also promise implied improved safety due to lowered dosages, however, any new agent must be carefully assessed for its clinical safety. Basic science discoveries often include initial in vitro cellular cytotoxicity studies, however these do not necessarily confirm safety when translated in vivo where materials are exposed to the numerous complexities encountered during circulation. As such, it is imperative that safety is considered at the design stage of new CAs. In this perspective, we aimed to highlight that self-assembly presents a promising strategy for enhancing MRI contrast agents by synergistically improving both T_1_ and T_2_ relaxation, increasing local concentration, and enabling multifunctional imaging capabilities. Although this approach offers exciting potential for next-generation imaging, several key challenges must be addressed to ensure its clinical applicability. In some cases, self-assembly may have the opposite effect, where aggregation-induced interactions lead to quenching rather than enhancement. For example, clustering of T_1_ agents (e.g., Gd^3+^-based) can reduce water accessibility, ultimately lowering relaxivity instead of improving MRI contrast performance. Additionally, uncontrolled aggregation and stability issues may result in structures exceeding the optimal nanoscale, leading to unpredictable biodistribution, altered pharmacokinetics, and clearance complications. Potential toxicity concerns arise from the instability of self-assembled structures, which may release high local concentrations of metal ions (e.g., Gd^3+^ or Fe^3+^) in vivo, posing safety risks. Moreover, larger assembled structures and particles may pose a greater risk of metal ion dissociation, due to their structural (in)­stability, or other toxicity concerns, potentially impacting safety. Another challenge is the unintended T_1_-to-T_2_ switching in iron oxide nanoparticles, where improper self-assembly can alter contrast behavior and affect imaging outcomes. Lastly, ensuring batch-to-batch reproducibility in the fabrication of self-assembled contrast agents remains a critical hurdle, especially when using stimuli-responsive or dynamic assembly mechanisms.

Currently, the majority of MRI CAs primarily serve as extracellular and blood pool agents. However, with the increasing importance of cellular imaging, future innovations are expected to prioritize intracellular imaging. The ideal imaging methods should highlight high signal specificity and achieve the highest possible contrast-to-noise ratio, while maintaining accuracy and temporal resolution. Suppression of unwanted signals or their avoidance should be a primary consideration in these developments. There is a noticeable shift toward intracellular self-assembly of MRI CAs, triggered by stimuli at targeted sites. Introducing biological vectors, such as monoclonal antibodies or oligopeptides for targeting CAs, holds great promise in medicinal and molecular biology practices. This approach offers increased efficiency and attractive prospects for imaging.

Assembling T_1_ and T_2_ CAs together can produce T_1_-T_2_ dual agents, while reversible assembly processes can lead to the development of T_2_-to-T_1_ and T_1_-to-T_2_ switchable contrast, offering an exciting evolution in clinical MRI practices which has, as yet, mostly continued to employ the traditional CAs and imaging practices from the early invention of MRI. Moreover, (bio)­activatable imaging probes sensitive to factors such as pH, light, or enzymes offer exciting opportunities for targeted tumor imaging and theranostic applications. These probes enable real-time monitoring and precise diagnosis, paving the way for personalized medicine approaches in oncology and beyond. The increased interest in multimodal probes capable of integrating various imaging modalities, such as MRI with optical/fluorescence, CT, SPECT, PET, among others, will further boost clinical prospects.

Even though there have been significant advances in creating and using self-assembly to prepare enhanced MRI CAs, many potential applications are still in the experimental phase. One of the notable reasons is that given the complex relationship between these parameters, the clinical success of MRI CAs involves more than simply tuning *r*_2_, *r*_1_, or *r*_2_/*r*_1_ ratios. Translation to clinical use requires practical considerations, including long-term storage, biological interactions, careful optimization of pharmacokinetics and circulation time in the body, imaging timing, large-scale synthesis, and cost. So far, a comprehensive understanding of the interplay between MRI, self-assembly, and biological systems is still incomplete. More systematic and thorough reporting, especially of adverse effects, which are often neglected, may provide greater insights into further development of MRI CAs. Engaging with clinicians during the early stages of design is also essential, as it provides critical insight into clinical needs, practical considerations for implementation and clinical workflows, technical acquisition, and patient acceptability. Incorporating this input early in the development process is crucial for ensuring clinical relevance and successful translation. It is notable that the translational trajectories of the different classes of agent remain distinct, with clinical challenges associated with nanoparticle-based systems (in particular iron oxides) historically hindering their widespread clinical adoption, despite promising performance.

Another significant challenge is the lack of standardized protocols for synthesizing ‘model particles’ that can be produced and surface-modified using standard approaches to ensure the final yield particles are reproducible and feature reliable stable properties. Similarly, creating uniform nanoparticle assemblies, especially for intracellular self-assembly, is still a significant challenge, where even the most rigorous strategies may still rely on a measure of serendipity. To improve consistency and scalability, more mechanistic studies are essential to advance the synthesis of uniformly assembled nanoparticles. Standardizing these protocols would minimize variations in singular particles and enable more consistent comparisons of results across different research groups/facilities. Similarly, a key challenge in evaluating MRI CAs is the difficulty of making conclusive comparisons across studies due to variations in experimental conditions, including field strength, temperature, and ligand structures. This lack of standardization complicates the direct attribution of relaxivity enhancement to specific factors. To advance the field, it would be beneficial for the research community to establish more consistent experimental protocols or reporting standards, enabling more meaningful comparisons and guiding the rational design of next-generation CAs. In particular, in order to fully understand properties and how we can advance the field further, in-depth analysis is required, for example using NMRD, which allows unpicking of the influence of different important relaxation parameters. The scientific community involved in MRI CAs for biomedical applications should collectively dedicate substantial efforts toward developing these standardized protocols. Such protocols would not only facilitate more meaningful comparisons of data but also play a pivotal role in translating magnetic nanoparticles from the bench to the clinic.
